# Orthostatic Hypotension and Urine Specific Gravity Among Collegiate Athletes

**DOI:** 10.7759/cureus.8792

**Published:** 2020-06-24

**Authors:** Sara K Arena, Emily Ellis, Carly Maas, Alex Pieters, Amy Quinnan, Rachel Schlagel, Tamara Hew-Butler

**Affiliations:** 1 Physical Therapy, Oakland University, Rochester, USA; 2 College of Education, Exercise and Sport Science, Wayne State University, Detroit, USA

**Keywords:** orthostatic hypotension, urine specific gravity, blood pressure, athletes

## Abstract

Introduction

The purpose of this study is to describe orthostatic blood pressure (BP) and urine specific gravity (USG) among collegiate athletes and then to examine if correlations between these variables could support use of orthostatic hypotension (OH) measures to screen for dehydration.

Methods

A prospective observational study was performed using a sample of convenience of collegiate athletes. Athlete’s sex and sport were recorded in addition to height, weight, seated and standing BP and USG measured at a pre- and post-season encounter. An OH response was defined as either the systolic BP decreasing ≥ 15 mmHg or the diastolic BP decreasing ≥ 7 mmHg when transitioning from sit to stand. The USG was considered positive for dehydration if >1.020. Descriptive statistics, pairwise t-tests, and the Spearman version of the correlation coefficient were used with statistical significance set at p < 0.05.

Results

Eighty athletes met inclusion criteria. Six athletes had an OH response during pre-season and three during post-season. Increased frequencies of athletes testing positive for dehydration were identified during the post-season compared to pre-season measures. No significant association was identified between OH and elevated USG. A secondary analysis identified significant associations between athletes with increased height and OH responses and correlations between higher BP and USG.

Conclusion

This study identified collegiate athletes with pre- and post-season OH as well as athletes with USG measures meeting the threshold for dehydration. While no correlation between OH and USG was identified, findings suggest screening of both BP and hydration status among collegiate athletes may be warranted.

## Introduction

Screening and assessment of vital signs is crucial in physical therapy practice to safely manage an individual’s medical status and prescribe exercise [1]. More specifically, blood pressure (BP) measurement is an essential screening tool for healthcare professionals, as abnormal readings often present without symptoms [2]. Prior evidence supports the fact that even populations considered generally healthy, such as collegiate athletes, may have elevated BP during both pre- and post-season assessments [3]. While there is evidence describing elevated BP in collegiate athletes, there is no evidence examining the presence of orthostatic hypotension (OH), which presents as a reduction in BP when transitioning to standing from sitting or lying down positions [4].

Orthostatic hypotension has traditionally been defined as a decrease in an individual's systolic BP of greater than or equal to 20 mmHg or a decrease in diastolic BP of greater than or equal to 10 mmHg when transitioning from either supine to sitting, sitting to standing, or supine to standing [4]. More recently, Shaw et al. recommended a drop in systolic of greater than or equal to 15 mmHg and diastolic of greater than or equal to 7 mmHg during a sit to stand transition to have improved sensitivity and specificity [5]. Although many aspects of OH are yet to be understood, OH has been identified as a risk factor for cardiovascular disease and mortality [6,7]. Though there is variability in the causation for OH, a hypohydrated or dehydrated state has been suggested as a possible underlying factor [4,8].

Kavouras reports urinary indices such as urine osmolality, urine specific gravity (USG), and urine conductivity and color, along with changes in body weight appear to provide the most accurate and sensitive evidence in monitoring hydration status [9]. Urine specific gravity is a laboratory test that utilizes either a refractometer or urine strip to compare the density of particles in urine compared to water [10]. Measurement of USG has been reported as an inexpensive, quick, and valid method to measure hydration status in a laboratory setting; however, it is not commonly used in a physical therapist (PT) practice [9,11]. While normal values of USG vary by institution, reference ranges generally fall between 1.005-1.030, with higher values indicating states of volume loss such as hypohydration or dehydration [12]. The American College of Sports Medicine (ACSM) defines dehydration as USG greater than 1.020 [13].

Hydration status in athletes is important to monitor before, during, and after activity, as dehydration has been observed to have a negative impact on athletic performance, heat dissipation, and overall health [14-16]. While urine indices such as USG are not routinely performed as a component of physical therapy care, PTs are well equipped to use orthostatic BP measures as a screening test and measure. Therefore, it seems plausible that if there is evidence to support that OH BP responses in athletes correlate to USG measures meeting cut points of hypohydration or dehydration, then PTs could be instrumental in identifying a sub-optimal hydration status and providing education to athletes under their care. Currently, there is limited evidence supporting a correlation between these two measures in the collegiate athlete population. Therefore, the purpose of this study is to describe orthostatic BP and USG among collegiate athletes and then to examine if correlations between these variables could support use of OH measures to screen for dehydration.

## Materials and methods

Research design

Following Oakland University Institutional Review Board (IRB) approval, National Collegiate Athletic Association (NCAA) Division I athletes from one university in Southeast Michigan were invited to participate in a prospective observational study using a sample of convenience.

Sampling criteria

All 2015-2016 athletes from the men’s and women’s basketball, men’s and women’s cross country, women’s soccer and men’s and women’s swimming/diving rosters were invited to participate. Athletes were informed of the study by a team representative or coach.

Protocol

After each athlete was deemed healthy during a pre-season physical, informed consent was secured. All encounters occurred in the Prevention Research Center on the campus of Oakland University. Investigators recorded sex, age, sport, height, and weight. Prior to obtaining BP measures, self-reported responses to screening questions inclusive of time of most recent workouts, current illness, recent tobacco use, and current medication consumption with a focus toward BP altering prescriptions were recorded. Athletes who participated in exercise 30 minutes prior to the BP assessment, were consuming medications with BP altering effects, reported a current illness, or consumed any form of tobacco products within 48 hours prior to BP assessment were excluded from data analysis. Athletes were not provided instruction to limit food or fluid consumption prior to testing; therefore, this was not a controlled component of the study methods.

Two consecutive BP readings were measured on the right upper extremity in both a seated and standing position and then were averaged for both encounters. The first encounter coincided with the athlete’s pre-season training and the second with the completion of the team’s fall competitive season, approximately a four-month time interval. Measures were obtained on the same day of the week (Friday), but given variability in academic schedules athletes self-selected the time of day to have the measures performed. Athletes were excluded from analysis if BP assessments were not available from both a pre- and post-season encounter. All investigators were trained in the study protocol with evidence-based BP methodology as a component of the data collection preparation. Investigators’ accuracy of measure was assured through competency testing using a BP simulation training device. Auscultatory technique with an appropriately sized and calibrated American Diagnostic Company brand Aneroid Sphygmomanometer^TM^ (Hauppauge, NY) and a Littman brand Master Classic II or Electronic Model 3100 Stethoscope^TM^ (Maplewood, MN) was used to obtain all BP measures. Each athlete remained in a seated position for a minimum of five minutes prior to taking the two seated BP measures at a minimum interval of one minute apart. The seated BP was measured with the athlete’s back supported, feet uncrossed, and flat on the floor, and arm supported at the level of the right atria [17,18].

Following the seated BP measures, the athlete stood and then two standing BP measures were measured after one minute and three minutes of standing. The right upper extremity was supported at the level of the right atria using an elevating table to accommodate variabilities in athlete height during the standing measurements [17,18]. Seated BP measures were classified as either meeting the definition of normal, elevated, or stage 1 or 2 hypertension (HTN) using the criteria set forth by 2017 ACC/AHA/AAPA/ABC/ACPM/AGS/APhA/ASH/ASPC/NMA/PCNA Guideline for the Prevention, Detection, Evaluation, and Management of High Blood Pressure in Adults (Table 1) [18]. An OH response was defined as a decrease in systolic BP of greater than or equal to 15 mm Hg or a decrease in diastolic BP of greater than or equal to 7 mm Hg when the athlete moved from the seated to standing position [5]. Supine BP measures were not obtained.

**Table 1:** Blood pressure classification used in current study

Blood Pressure Classifications	Systolic Blood Pressure (mm Hg)	Diastolic Blood Pressure (mm Hg)
Normal	<120	<80
Elevated	120 to 129	<80
Stage 1 Hypertension	130-139	80-89
Stage 2 Hypertension	≥140	≥90

After obtaining a urine sample from each athlete, USG measures were obtained using a URS-10 CHEMSTRIPTM (Roche Diagnostics; Basel, Switzerland) inserted into an Uritek TC-101 urine reader (Tecodiagnostics, Anaheim CA). The athlete’s urine was classified as normal if less than or equal to 1.020 or meeting the definition of dehydration if greater than 1.020 consistent with the ACSM dehydration definition cutpoint [13].

Data analysis

Descriptive statistics reported athlete demographics, BP classifications in the seated position, OH BP responses, and USG. Pairwise t-tests determined differences in OH BP measures and USG overall and then by sex and height at both the pre- and post-season encounters. Additionally, prior evidence of elevated BP in collegiate athletes and elevations in BP brought about by dehydration, warranted a secondary analysis examining possible differences or similarities between seated BP measures and USG [3,14]. Spearman’s rank correlation coefficient test examined correlations when differences were identified. Statistical significance was set a priori at p < .05.

## Results

Athlete demographics

One hundred and twenty-seven collegiate athletes presented for pre-season assessment with 47 lost to attrition during the post-season assessment for unknown reasons. No athlete was excluded for having exercised in the prior 30 minutes, illness, consuming BP altering medications or tobacco. Of the 80 athletes ultimately meeting the inclusion criteria, 26 were male and 54 were female. Athletes represented women’s soccer 16.3% (n = 13), men’s basketball 12.5% (n = 10), women’s basketball 15.0% (n = 12), men’s cross country 12.5% (n = 10), women’s cross country 21.3% (n = 17), men’s swimming/diving 7.5% (n = 6) and women’s swimming/diving 15.0% (n = 12). No athlete identified pre-existing hypotension during the initial encounter. Table 2 provides height and weight results by sport and sex.

**Table 2:** Height and weight by sport and sex for the current study (n = 80) SD = standard deviation; cm = centimeters; kg = kilograms

Sport	Height in cm (SD)	Weight in kg (SD)
Men’s Basketball	193.3 (10.2)	93.4 (13.7)
Men’s Cross Country	179.3 (5.8)	68.0 (6.7)
Men’s Swimming	183.3 (7.4)	78.6 (4.5)
All Males	185.6 (10.2)	80.2 (14.8)
Women’s Soccer	170.1 (6.3)	65.2 (12.3)
Women’s Swimming	170.0 (5.7)	66.1 (5.0)
Women’s Cross Country	164.9 (6.1)	56.0 (5.0)
Women’s Basketball	174.2 (7.1)	73.3 (11.2)
All Females	169.3 (7.2)	64.3 (10.9)
All Athletes	174.6 (11.3)	69.4 (14.4)

Blood pressure classification

The athletes pre-season BP classifications were as follows: 73.8% (n = 59) normal BP, 15.0% (n = 12) elevated BP, 11.3% (n = 9) HTN stage 1 and no athletes met the criteria for stage 2 HTN. Post-season BP classifications were as follows: 73.8% (n = 59) normal BP, 7.5% (n = 6) elevated BP, 17.5% (n = 14) with HTN stage 1 and 1.3% (n = 1) with HTN stage 2. Table 3 details BP classifications results by sport.

**Table 3:** Blood pressure classification for athletes by sport HTN = Hypertension

Sport	Pre-Season Blood Pressure Classification % (n)				Post-Season Blood Pressure Classification % (n)			
	Normal	Elevated	Stage 1 HTN	Stage 2 HTN	Normal	Elevated	Stage 1 HTN	Stage 2 HTN
Men’s Basketball	30.0 (3)	20.0 (2)	50.0 (5)	0	20.0 (2)	20.0 (2)	60.0 (6)	0
Women’s Soccer	84.6 (11)	7.7 (1)	7.7 (1)	0	76.9 (10)	7.7 (1)	15.4 (2)	0
Women’s swimming	91.7 (11)	0	8.3 (1)	0	83.3 (10)	0	16.7 (2)	0
Men’s Cross Country	70.0 (7)	20.0 (2)	10.0 (1)	0	60.0 (6)	10.0 (1)	20.0 (2)	10.0 (1)
Women’s Cross Country	88.2 (15)	11.7 (2)	0	0	94.1 (16)	5.9 (1)	0	0
Men’s Swimming	33.3 (2)	66.7 (4)	0	0	83.3 (5)	0	16.7 (1)	0
Women’s Basketball	83.3 (10)	8.3 (1)	8.3 (1)	0	83.3 (10)	8.3 (1)	8.3 (1)	0

Orthostatic hypotension

Six athletes were identified as having an OH response in the pre-season assessment. All six athletes were male, one a member of the basketball team, two members of the cross country team and the other three were members of the swimming and diving team. A significant difference was identified in the occurrence of OH between males and females (0.0002) at the pre-season measure. Additionally, athletes with OH were identified to have significantly taller stature (p = 0.01), a higher seated systolic BP measure (p = 0.002) and a higher standing diastolic BP measure (p = 0.001) at the pre-season encounter than their counterparts without OH. A comparison of OH to USG found no statistically significance relationship (p = 0.42) at the pre-season encounter.

Two male athletes, one from the basketball team and one from the swimming and diving team, continued to have an OH response in the post-season measurement. Additionally, a female athlete from the basketball team presented with an OH response at the post-season measurement. No significant difference was identified in the occurrence of OH between males and females (0.20) at the post-season measure. Post-season analysis of a relationship between taller stature and OH continued to reveal a significant finding (p = 0.003); however, only the standing systolic BP measures reached a significant level (p = 0.01) among those with OH at the post-season encounter. Additionally, a comparison of OH to USG during the post-season encounter did not identify a significant relationship (p = 0.45).

Urine specific gravity

The mean USG of all athletes was 1.016 with a range of 1.005 to 1.030. Pre-season USG classifications were as follows: 91.0% (n = 71) met the criteria for classification as normal hydration and 9.0% (n = 7) met the definition of dehydrated. Post-season USG classifications were as follows: 69.6% (n = 55) meet the criteria for classification as normal hydration and 30.4% (n = 24) met the definition of dehydrated. Table 4 details USG results by sport, sex, and pre- and post-season measurement encounters.

**Table 4:** Urine specific gravity by sport for the current study (n = 80) USG = Urine specific gravity

Sport	Average Pre-season USG	Pre-season Normal % (n)	Pre-season Dehydrated % (n)	Average Post-season USG	Post-season Normal % (n)	Post-season Dehydrated % (n)
Men’s Basketball	1.018	80.0 (8)	20.0 (2)	1.018	80.0 (8)	20.0 (2)
Men’s Cross Country	1.018	88.9 (8)	11.1 (1)	1.022	50.0 (5)	50.0 (5)
Men’s Swimming	1.012	100.0 (6)	0	1.014	100.0 (5)	0
Men	1.016	88.0 (22)	12.0 (3)	1.019	72.0 (18)	28.0 (7)
Women’s Soccer	1.018	76.9 (10)	23.1 (3)	1.018	76.9 (10)	23.1 (3)
Women’s Swimming	1.014	100.0 (11)	0	1.017	75.0 (9)	25.0 (3)
Women’s Cross Country	1.015	100.0 (17)	0	1.016	70.6 (12)	29.4 (5)
Women’s Basketball	1.015	91.7 (11)	8.3 (1)	1.020	50.0 (6)	50.0 (6)
Women	1.015	92.5 (49)	7.5 (4)	1.018	68.5 (37)	31.5 (17)
Total	1.016	91.0 (71)	9.0 (7)	1.018	69.6 (55)	30.4 (24)

Correlation of urine specific gravity and elevated blood pressure measures

As was previously reported the comparison of OH to USG found no statistically significance relationship; therefore, correlation analysis of these relationships was not indicated. However, a secondary analysis revealed there was a significant correlation between pre-season standing diastolic BP and USG (r = 0.27, p = 0.02), post-season seated systolic BP (r = 0.35; p = 0.002), and post-season standing systolic BP (r = 0.30, p = 0.01). In other words, higher BP measures were correlated to higher USG measures. Table 5 details these correlations.

**Table 5:** Correlation between urine specific gravity and higher blood pressure measures *denotes variable reached level of significance (p < 0.05) r = correlation coefficient; p = p-value

Measurement encounter	Seated blood pressure measurement		Standing blood pressure measurement	
	Systolic	Diastolic	Systolic	Diastolic
Pre-season	(r = .04, p = .73)	(r = .22, p = .05)	(r = .03, p = .78)	(r = .27, p = .02)*
Post-season	(r = .35, p = .002)*	(r = .22, p = .05)	(r = .30, p = .01)*	(r = .16, p = .17)

## Discussion

The purpose of this study is to describe orthostatic BP and USG among collegiate athletes and then to examine if correlations between these variables could support use of OH measures to screen for dehydration. This study found OH among collegiate athletes, but not a correlation to the USG measures; however, further studies controlling for fluid and caffeine consumption are warranted. While this study did not examine causation and long-term impact of OH on this population, prior studies have reported a correlation between OH and an increased risk of mortality, stroke, and incidence of heart failure in an older population [19,20]. Additionally, when using USG ≥ 1.020 as an indicator of dehydration, athletes were identified with dehydration and the frequency increased at the post-season measurement. There is prior evidence to suggest dehydration results in decreased performance capacity, visual motor tracking, attention, and short-term memory loss [21]. Therefore, the negative impact of dehydration on sports performance and cognition should be considered and addressed by PTs through patient education with an intention to optimize the athlete’s functional and performance outcomes [21,22].

A relationship was not identified between the occurrence of OH and an elevated USG, suggesting the use of orthostatic BP measures may not be warranted to determine an athlete’s hydration status. There is evidence to suggest USG measures may only represent urine concentration at the level of the kidney and is not a true indicator of clinical dehydration [23]. It has been further implied that individuals with a dehydration diagnosis are generally hypovolemic and therefore, may be more likely to present with an associated lower BP [24]. Therefore, future iterations of this study may warrant inclusion of blood testing which could be utilized as a technique to measure blood volume [25]. This methodology may better quantify the hypovolemic status of an individual and could potentially identify correlation between an OH response to volume measures of dehydration which was not observed using the current study protocol.

Given this study did not find OH measures to be a useful tool in determining hydration status and many of the osmolality and hypovolemic blood markers are not easily accessible to PTs working with athletes, incorporation of more widely available screening techniques into routine PT practice could be beneficial. Additionally, models that provide athletes instruction to self-monitor long term are warranted. The WUT model provides one such monitoring technique [26]. This model uses three markers of hydration: weight (W), urine (U), and thirst (T). While the presence of one marker is not highly suggestive of dehydration, the collective finding of loss of body weight of 1% in a day, change in urine color and frequency, and the presence of thirst as an indicator of dehydration would suggest an increased likelihood of dehydration.

A relationship between OH and individuals with a taller stature is in congruence with prior evidence from a study examining an older adult population [27]. However, height bias related to sex should be considered within the scope of this study findings given pre-season OH responses were only identified in males. It is notable that collegiate athletes have been reported to have average heights above those reported for their non-athlete counterparts [28]. Therefore, routine screening for OH may have significant value in identifying OH measure in populations known to have a taller stature inclusive of collegiate athletes. Furthermore, routine BP screening is further supported in The Guide to Physical Therapist Practice as the measurement findings may be useful in detecting OH or elevated BP in all individuals under the care of PTs [29].

Study limitations

A small number of athletes identified as having OH which limited comparisons and correlations. Additionally, associated OH symptoms were not specifically recorded during data collection. Furthermore, the use of only the sit to stand transitional movement may have limited the frequency of OH responses results which may have otherwise been observed by inclusion of supine measures. Finally, USG was the single measure of dehydration and may have only represented urine concentration at the kidneys and not clinical dehydration.

Future research

An investigation of OH and dehydration findings with intention to larger samples by sport and sex would be helpful to identify any difference among these variables. Future research inclusive of symptom provocation in the presence of OH response is warranted. The addition of a multifactorial assessment for dehydration, including symptoms, skin turgor, or other measures of hypovolemia would be beneficial next steps in future research. Furthermore, the authors suggest the inclusion of the supine position in the OH measures in future investigations. Finally, research design aimed at examining causative factors for the presence of OH among collegiate athletes of taller stature is warranted.

## Conclusions

This study identified collegiate athletes with pre- and post-season OH (n = 6 and n = 2, respectively) as well as athletes with USG measures meeting the ACSM threshold for dehydration. Notably, while seven athletes were identified as having dehydration at the pre-season encounter this number increased to 24 at post-season. No correlation between OH and USG was identified. Given a three-fold increase in dehydration over the course of the athletes competitive season, PTs should be mindful to assess for this condition when providing care to this population. Furthermore, the secondary finding of BP measures which met the criterion for classification as elevated, stage 1, and stage 2 HTN supports prior recommendations to measure an athlete's BP as a primary prevention strategy throughout their competitive career. Routine BP screening may be useful in detecting OH or elevated BP in all individuals under the care of PT including seemingly healthy collegiate athletes.
